# A randomized open label trial of tamoxifen combined with amphotericin B and fluconazole for cryptococcal meningitis.

**DOI:** 10.12688/wellcomeopenres.15010.1

**Published:** 2019-01-22

**Authors:** Nguyen Thi Thuy Ngan, Nguyen Thi Hoang Mai, Nguyen Le Nhu Tung, Nguyen Phu Huong Lan, Luong Thi Hue Tai, Nguyen Hoan Phu, Nguyen Van Vinh Chau, Tran Quang Binh, Le Quoc Hung, Justin Beardsley, Nicholas White, David Lalloo, Damian Krysan, William Hope, Ronald Geskus, Marcel Wolbers, Le Thanh Hoang Nhat, Guy Thwaites, Evelyne Kestelyn, Jeremy Day

**Affiliations:** 1 Oxford University Clinical Research Unit, University of Oxford, Ho Chi Minh City, Vietnam; 2 Dept of Tropical Medicine, Cho Ray Hospital, Ho Chi Minh City, Vietnam; 3 Hospital for Tropical Diseases, Ho Chi Minh City, Vietnam; 4 Cente for Tropical Medicine, University of Oxford, Oxford, UK; 5 Liverpool School of Tropical Medicine, Liverpool, UK; 6 Depatrment of Pediatrics and Microbiology/Immunology, University of Iowa, Iowa City, USA; 7 Molecular and Clinical Pharmacology, Universitly of Liverpool, Liverpool, UK

**Keywords:** Tamoxifen, fluconazole, amphotericin B, antifungal therapy, cryptococcal meningitis, Crytococcus, drug re-purposing

## Abstract

**Background**: Cryptococcal meningitis is a leading cause of death in HIV-infected patients. International treatment guidelines recommend induction therapy with amphotericin B and flucytosine. This antifungal combination is most effective, but unfortunately flucytosine is expensive and unavailable where the burden of disease is greatest. Where unavailable, guidelines recommend treatment with amphotericin and fluconazole, but this is less effective, with mortality rates of 40-50%. Faster rates of clearance of yeast from cerebrospinal fluid (CSF) are associated with better outcomes - improving the potency of antifungal therapy is likely to be an effective strategy to improve survival. Tamoxifen, a selective estrogen receptor modulator used to treat breast cancer, has anti-cryptococcal activity, appearing synergistic when combined *in vitro *with amphotericin, and fungicidal when combined with fluconazole. It is concentrated in the brain and macrophages, off-patent, cheap and widely available. We designed a randomized trial to deliver initial efficacy and safety data for tamoxifen combined with amphotericin and fluconazole.

**Method**: A phase II, open-label, randomized (1:1) controlled trial of tamoxifen (300mg/day) combined with amphotericin (1mg/kg/day) and fluconazole (800mg/day) for the first 2 weeks therapy for HIV infected or uninfected adults with cryptococcal meningitis. The study recruits at Cho Ray Hospital and the Hospital for Tropical Diseases, Ho Chi Minh City, Vietnam. The primary end point is Early Fungicidal Activity (EFA-the rate of yeast clearance from CSF), over the first two weeks of treatment. 50 patients will be recruited providing ≈80% and 90% power to detect a difference in the EFA of -0.11 or -0.13 log10CFU/ml/day, respectively.

**Discussion:** The results of the study will inform the decision to proceed to a larger trial powered to mortality. The size of effect detectable has previously been associated with reduced mortality from this devastating disease. Particular side effects of interest include QT prolongation.

**Trial registration**: Clinicaltrials.gov NCT03112031 (11/04/2017)

## Background

Worldwide cryptococcal meningitis results in approximately 625 000 deaths each year, most occurring within 3 months of diagnosis ^1^. It is the leading cause of death in HIV patients in Asia and Africa affecting 3.2% of the HIV infected population per year ^1^. Despite improvements in access to HIV care, the WHO estimates that even if 80% access to HIV treatment is achieved, there will be 6.5 million AIDS deaths p.a. by 2030 ^2^. Thus, CM is likely to remain a significant health burden for the foreseeable future. While cryptococcal meningitis predominantly occurs in HIV infected patients, patients with other forms of immunosuppression are also affected ^3, 4^. Furthermore, in Viet Nam we see disease in apparently immunocompetent patients (N = 30 each year at the Hospital for Tropical Diseases and Cho Ray Hospital) due to infection with *Cryptococcus neoformans* var. *grubii*, or, more rarely, *C. gattii* ^5^.

There has been no major advance in the treatment of cryptococcal meningitis since the 1980’s, and the 90 day case fatality rate of cryptococcal meningitis remains unacceptably high, estimated at 55% in Asia and 70% in Africa ^1^. The mainstays of induction therapy are drugs that are over 50 years old – amphotericin B and flucytosine - although these are often poorly available where disease burden is highest ^6, 7^. While amphotericin monotherapy is undoubtedly superior to fluconazole monotherapy, amphotericin combination therapy has only recently been shown to reduce mortality when compared with amphotericin monotherapy in a randomized controlled trial in Vietnam ^8^. In this study, in Vietnamese patients receiving gold standard treatment with amphotericin and flucytosine, the death rate at 10 weeks and 6 months was 31% and 35%. This compared with 33% and 46% in patients receiving amphotericin-fluconazole induction therapy (the current WHO guideline), and was significantly better than in patients receiving prolonged amphotericin monotherapy, where the death rates were 44% and 54% respectively ^8^. A biological explanation for these better outcomes is that the amphotericin-flucytosine combination has increased sterilising power – it results in significantly more rapid clearance of yeast from cerebrospinal fluid than amphotericin monotherapy ^8^. Consistent with this, the recent multi-country trial of adjunctive dexamethasone in HIV associated cryptococcal meningitis has shown that this immune modulating treatment is associated with both reduced rates of clearance of yeast from CSF and worse clinical outcomes ^9^.

Therefore, it seems that the rate of clearance of yeast from CSF is an important determinant of outcome from cryptococcal meningitis, and that improving the potency of antifungal therapy is likely to result in improved outcomes. Currently, flucytosine is unaffordable (costs of the most recently developed generic version are 15 000 USD/week), unavailable outside of Europe and the US, and has significant toxicities which can be difficult to manage in resource-poor settings. Therefore it is not currently a viable solution for patients where the vast burden of cryptococcal disease occurs ^10^. Instead, most patients worldwide are treated with amphotericin and fluconazole, as per WHO guidelines ^7^. There is a pressing need to develop affordable, safe and practicable antifungal treatment regimens with greater sterilising power.

### Current Treatment recommendations for CM

Successful treatment of CM depends upon effective anti-fungal therapy and successful management of complications, notably raised intracranial pressure. Antifungal treatment schedules for cryptococcal meningitis are not globally uniform but are affected by drug availability, costs and human resources. The Infectious Diseases Society of America convenes an international panel to draw up treatment guidelines, most recently published in 2010, and the WHO published guidelines aimed at management in resource-poor countries in 2011 ^6, 7^. These conform closely to the IDSA guidelines. Treatment generally consists of a period of induction therapy using high dose or combination antifungal therapy (usually for 2 weeks), followed by a period of consolidation therapy of 8 weeks with fluconazole. After this time, provided the patient has responded to treatment, secondary prophylaxis using lower dose fluconazole is given to prevent disease relapse. It is generally considered safe to stop secondary prophylaxis if ARV therapy has resulted in suppression of the plasma viral load and there has been immune reconstitution with recovery of the CD4 cell count to > 100 cells/uL for at least 6 months.

Consistent with the local practices and the WHO and IDSA guidelines, in this study all patients in the control arm will receive anti-fungal therapy consisting of amphotericin B (1mg/kg/day) combined with fluconazole 800mg/day for 2 weeks, followed by fluconazole 800mg/day for a further 8 weeks before switching to secondary prophylaxis ^6, 7^.

## Rationale for a trial of tamoxifen

### Current indications

Tamoxifen is an off-patent, widely available (licensed in Vietnam) and affordable selective oestrogen receptor modulator (SERM) ^11^. Its initial indication was the treatment of breast cancer. There are many thousands of patient years’ experience of its use for this disease, where it has proved to be a safe, effective, and tolerable long term therapy. In addition to its effects against breast cancer, Tamoxifen, in higher doses, also has activity against other tumours including glioblastoma multiforme (GBM), desmoid tumours, lung and prostate cancer ^12– 16^.

### Antifungal activity

In addition to its anti-cancer effects, Tamoxifen has been known to have activity against yeasts for several years, this first being identified against the model yeast *Saccharomyces cerevisiae* in 1989, and subsequently demonstrated against *C. albicans* ^17– 19^. In 2009 the first reports of activity against *Cryptococcus neoformans* were described ^20^. The antifungal activity is of particular interest for the following reasons:

1. *In vitro*, the combination of Tamoxifen with amphotericin is **synergistic**. This contrasts with the current most effective antifungal therapy – amphotericin and flucytosine – where the effects of each drug are simply additive ^11^. A synergistic antifungal combination offers the opportunity to significantly improve CSF sterilisation and survival.
2. In the mouse model of cryptococcosis, the combination of Tamoxifen with fluconazole is **fungicidal** ^11, 21^. No other antifungal drug clearly has a fungicidal effect *in vivo*. The lack of fungicidal effect of conventional antifungal therapies may in part explain the slow cerebrospinal fluid (CSF) sterilisation and high death rates seen in cryptococcal disease.
3. Tamoxifen is **concentrated in brain tissue** ^22, 23^. This is the main site of cryptococcal disease. Current antifungal drugs result in relatively rapid sterilisation of blood. However, they are much less effective at sterilising the central nervous system, with only approximately 50% of patients having sterile cerebrospinal fluid after 2 weeks of conventional treatment ^8^.
4. Tamoxifen is **concentrated in the macrophage phagosome** ^11^. In cryptococcal meningitis, yeast cells are engulfed by macrophages, but can survive and even reproduce within them. Unlike Tamoxifen, conventional antifungal drugs cannot penetrate the macrophage phagosome, and this therefore represents a site where yeast cells may be relatively protected from treatment.

A drug combination with both fungicidal and synergistic activity, that is concentrated in the central nervous system and within macrophages, is an exciting prospect for cryptococcal disease, and has the potential to significantly improve outcomes.

### Data from Vietnam

*In vitro* and *in vivo* experimental data concerning the efficacy of Tamoxifen have been derived from experiments using the *C. neoformans* type strain H99. The Minimum Inhibitory Concentration (MIC) of Tamoxifen for H99 is 8-16ug/ml. To ensure applicability in Vietnam, we have determined the MIC of Tamoxifen for 30 clinical isolates of *C. neoformans* and *C. gattii f*rom Vietnamese patients with cryptococcal meningitis and confirmed it to be between 2-16ug/ml ( *unpublished data*). We have also demonstrated that synergy with amphotericin B *in vitro* is seen for the majority of strains tested. Subsequently we have performed 3D chequerboard testing of the triple combination of amphotericin with fluconazole and Tamoxifen, and have shown that there is also synergy between the 3 drugs in triple combination, and moreover that a synergistic interaction is also present for the few strains in which we failed to demonstrate synergy with the 2 drug combination. We have also demonstrated that there is no synergistic interaction *in vitro* when Tamoxifen is combined with flucytosine.

### Mechanism of action

Tamoxifen is a selective estrogen modulating drug (SERM) that has both pro- and anti-estrogen effects. In breast cancer, it inhibits growth of (cancerous) breast tissue through modulating the expression of estrogen dependent genes and the subsequent expression of growth promoting signals. In *Cryptococcus*, Tamoxifen binds to calmodulin and calmodulin-like protein, preventing the activation of calcineurin ^11, 20^. The calmodulin-calcineurin pathway is key in intra-cellular signalling and in controlling the response of *Cryptococcus* to stress, such as that encountered following phagocytosis by macrophages.

### Pharmacology and dosing

Tamoxifen has excellent dose-independent oral bioavailability (approximately 100%) with minimal first-pass metabolism ^24^. The half-life is 5–7 days. Tamoxifen’s SERM activity requires serum concentrations ~100 ng/mL. This is achieved with doses of 20 – 60 mg/day, and for breast cancer treatment at this dose is recommended for 5–10 years ^25^. At these doses, brain tissue concentrations are 40-100-fold higher (4-10 µg/mL), than in serum ^23^. Higher doses of 240 – 500mg/day have been studied in clinical trials of treatment for CNS tumours, desmoid tumours and lung cancer and are well tolerated, safely achieving serum concentrations of 2-8ug/mL ^12– 14, 26– 28^. Treatment administration regimens at these doses vary: month-long cycles of 300mg/day are given for desmoid tumours in children ^28^; doses of 120mg/m ^2^ BID have been given for up to 1 year and well tolerated in patients with glioblastoma multiforme ^27^. Such treatment regimens establish Tamoxifen serum concentrations around the *C. neoformans* MIC and, will achieve brain concentrations 40-100-fold higher, suggesting levels sufficient to treat CM will be achieved using oral dosing in humans ^23^. Moreover, uniquely compared with other antifungal drugs, Tamoxifen is further concentrated in macrophage phagosomes – an important site of *C. neoformans* replication ^11^.

### Adverse effects

Tamoxifen is a well-tolerated drug. Adverse effects must be considered in the context of the severity of the disease being treated. Survival rates for breast cancer, GBM and cryptococcal meningitis are illustrated in Table 1. The beneficial effects of Tamoxifen in women diagnosed with breast cancer are considered to considerably outweigh the potential adverse effects of Tamoxifen.

**Table 1.** Survival rates: breast cancer, glioblastoma and cryptococcal meningitis.

Condition	Survival Metric	Survival rate *	Tamoxifen dose used
Breast Cancer	1 year survival	95%	20–40mg/day
Glioblastoma multiforme	2 year survival rate	66%	300mg/day
Small cell lung cancer	5 year survival rate	8 – 31%	300–500mg/day
Cryptococcal meningitis	6 month survival rate	45 – 55%	Suggested 300mg/day

*Sources: http://www.cancerresearchuk.org/about-cancer/type, American Cancer Society, Beardsley *et al.*, NEJM 2014, Park *et al*., 2009. Note, survival rates are overall survival rates for the condition stated, and not specifically for Tamoxifen containing regimens.

## Headaches, nausea and vomiting

Data from randomised controlled trials suggest that these symptoms are no more common than in patients receiving placebo ^29^.

## Hot flushes and impaired libido

These symptoms are likely mediated through oestrogen modulation, can occur acutely, and are reversible when treatment stops.

## Pulmonary embolism, cataracts and endometrial cancer

Data from trials of prophylactic treatment for breast cancer suggest that long term use is associated with an increased risk of pulmonary embolism (Relative Risk (RR) = 3.01, 95% Confidence Interval (CI): 1.15 to 9.27), cataracts (RR = 1.13, 95% CI: 1.00 to 1.28)and endometrial cancer (RR = 2.48, 95% CI: 1.27 to 4.92, ^30^. However, the actual rate of these events is very low (0.75 per 1000 patient years, 25 per 1000 patient years and 2.2 per 1000 patient years respectively) ^29, 30^. Thromboembolic events occurred an average of 19 months after starting treatment, and none within the first 2 months ^30^. The risk of these events with short course treatment is much lower. It is not considered worthwhile to screen for factor V Leiden prior to treatment with Tamoxifen unless there is a family or personal history of previous thromboembolic disease. The increased risk of endometrial cancer is likely due to chronic modulation of oestrogen, is related to duration of use, and clearly does not exist for male patients.

## Teratogenicity

Tamoxifen is teratogenic and should not be taken by pregnant women. Fluconazole is also a teratogenic drug, and pregnant women are not eligible to enter this study. Women of child-bearing potential will be screened at study entry for pregnancy with a urine pregnancy test. All study participants will be advised to use barrier contraception until at least 3 months after study end.

## Prolongation of the QT interval

Tamoxifen can result in prolongation of the QT interval of cardiac depolarisation ^16, 31^. QT prolongation is a recognised side effect of a number of drugs including some antimicrobials, anti-arrhythmic drugs and psychoactive drugs ^32^. For some drugs, QT prolongation is associated with a risk of developing ventricular arrhythmias, particularly Torsades de Pointes (TdP). While this is a rare event, if it occurs, TdP can be life-threatening. However, not all drugs that prolong the QT increase this risk – some drugs that prolong the QT actually have anti-arrhythmic effects. Whether a drug increases the risk of TdP or not is believed to be determined by the mechanism of inhibition of ventricular repolarisation, and the duration of prolongation of the QT interval. TdP is treated with removal of the stimulus, intravenous magnesium, correction of electrolyte abnormalities, and DC cardioversion or overdrive pacing.

The mechanism of QT interval prolongation varies with different drugs and can be due to modulation of single or multiple currents involved in cardiac depolarisation ^32, 33^. Data suggest that modulation of a single current may be more likely to result in arrhythmias than QT prolongation due to modulation of multiple currents/channels ^34^. The mechanism of QT prolongation with Tamoxifen in humans is unknown. In animals there is evidence that the block is multi-channel, due to both inhibition of the I _KR_ and I _Ca_ channels, and may be therefore less likely to cause arrhythmia ^35– 37^. Supportive of a multichannel block mechanism for Tamoxifen is the fact that there are no case reports of TdP with Tamoxifen use, despite its extensive prescription.

Fluconazole can also result in QT prolongation. This is believed to be mediated through modulation of the I _KR_ current of the cardiac depolarisation cycle, but data are lacking concerning whether there are also effects on other currents ^38^. TdP has been reported in patients receiving fluconazole, although again, this is a rare event ^39, 40^.

The effect of combining drugs that prolong the QT may depend upon the total number of mechanisms through which the QT is prolonged, and combining such drugs will not necessarily increase the risk of TdP ^41, 42^. In this study, to mitigate any risk of prolonged QT, all patients will have meticulous monitoring and correction of electrolyte disturbances including potassium, calcium and magnesium, and twice daily ECG monitoring. The QT interval will be manually determined by measuring the interval in 3 limb and 3 chest leads, and determining the median (machine derived estimates of the QT interval are unreliable) ^43^. The use of manual estimate of the QT has been shown to have similar performance to high resolution holter ECG monitoring ^43^. Management of QTc prolongation will be in line with the recommendations of the American Heart Association ^32^. The QT interval will be corrected (QTc) for rate using the Framingham formula. Where the QTc is found to be >500ms, the status of the patients K, Ca and Mg levels will be reviewed, corrected if necessary, and the ECG repeated before the next dose of Tamoxifen is due. If the QTc remains >500ms, the next due dose of Tamoxifen will be omitted until it is less than 500ms. When the QTc is less than 500ms, Tamoxifen will be restarted.

## Other effects

An early trial of high dose Tamoxifen (equivalent to >450mg/day) in patients with epithelial tumours reported a sense of light-headedness and unsteady gait in a proportion of patients, which resolved within 2–5 days of stopping the drug ^26^. Tamoxifen does not have any expected haematological or hepatic side effects.

### Drug interactions

Tamoxifen enhances the effects of warfarin and similar medications, which should be avoided. It is metabolised via the cytochrome p450 pathway and may elevate the levels of drugs metabolized through this pathway, including fluconazole. Pharmacokinetic sampling will define whether there is any measurable effect of Tamoxifen on fluconazole levels in this study. For all patients the drug history will be ascertained at admission through the medical clerking and through examination of any accompanying medication where the patient is unsure of the nature of previously prescribed treatment.

## Study aims

### Primary aim

To determine whether adding Tamoxifen to standard antifungal therapy increases the rate of clearance of yeast from cerebrospinal fluid in patients with cryptococcal meningitis. The rate of clearance will be defined as the Early Fungicidal Activity (EFA), as previously described ^8, 9^.

### Secondary aims

The secondary aims are to

- demonstrate the proof of concept that Tamoxifen can be safely administered with amphotericin and fluconazole to generate preliminary data that will support a larger phase 3 trial of Tamoxifen in cryptococcal disease
- determine the effects on survival and disability at 10 weeks following study treatment initiation,
- define the pharmacokinetics of amphotericin, fluconazole and Tamoxifen in blood and CSF
- better understand the pathogenesis of cryptococcal meningitis through defining the species, subtype, drug susceptibility and in situ gene expression of the infecting *Cryptococcus* and host response

## Endpoints

### Primary endpoint

The primary outcome is Early Fungicidal Activity (EFA), i.e. the rate of clearance of yeast from cerebrospinal fluid, over the first 2 weeks following randomisation. Repeated lumbar punctures are part of routine care for cryptococcal meningitis in order to identify and manage raised intracranial pressure and to determine the response to treatment. In this trial, lumbar punctures are scheduled on days 1, 3, 7, 14, and additionally as clinically indicated. Whenever a lumbar puncture is performed we will determine the amount of viable yeast in CSF through culture. Based on the patients’ longitudinal quantitative yeast count measurements, EFA will be determined as described in the statistics section. Higher EFAs have been associated with better outcomes (survival and disability) in Vietnamese patients with cryptococcal meningitis ^8, 9^.

### Secondary endpoints

***Survival until 10 weeks after randomization.*** International treatment guidelines recommend 10 weeks of high dose antifungal therapy for cryptococcal meningitis – an initial phase of amphotericin based induction therapy for 2 weeks followed by 8 weeks of moderate to high dose fluconazole. The rate of survival until this 10 week period of therapy is completed is a frequent endpoint in trials of treatment for cryptococcal meningitis ^8, 9, 44^. Improvements in EFA over the first 2 weeks of treatment have been associated with improved survival at 10 weeks. Therefore survival until 10 weeks after randomization will be a secondary endpoint in this study.

***Disability at 10 weeks.*** Disability is an expected consequence of cryptococcal meningitis, including blindness, deafness and other focal neurological deficits. In addition to improving the sterilizing power of antifungal therapy, Tamoxifen may also reduce the rates of disability in survivors. Neurological disability will be assessed using the modified Rankin score and the Two Simple Questions and classified as good, intermediate, severe disability, or death, as previously described ^45^.

***Adverse events.*** The proportion of patients with any grade 3 or 4 adverse event, serious adverse event, or unexpected serious adverse event will be compared between treatment groups.

***Rate of IRIS until 10 weeks (in HIV infected patients only).*** We will model the rate of IRIS over time with a cause-specific hazards model taking into account the competing risk of prior death. CM-related IRIS will be defined as per the recent proposed definition ^46^.

***Rate of Cryptococcal meningitis relapse.*** A pragmatic definition of relapse will be used. This is defined as either intensification of antifungal therapy above that according to the study antifungal schedule, or readmission for treatment of cryptococcal disease.

***QT prolongation.*** Prolongation of the QT interval is a potential side-effect of both Tamoxifen and fluconazole, although it is not clear that either drug increases the risk of Torsade de Pointes, a potentially life-threatening arrhythmia. The QT interval will be estimated manually from 3 chest and 3 limb leads from a high resolution (50mm/sec) 12-lead ECG. The median value will be determined and used to calculate the corrected QT interval (QTc) using Framinghams’ formula.

***Visual deficit at 10 weeks.*** Visual deficit occurs in 5–40% of patients with cryptococcal meningitis depending upon underlying immune status ^5, 8^. The pathogenesis is unclear. We will compare the incidence of blindness and other visual deficit between treatment groups. Visual deficit will be assessed using a simple 6 point scale, as used in previous studies (see Table 3).

***Time to new neurological event or death until 10 weeks.*** A neurological event is defined as a fall in Glasgow coma score by ≥2 points for ≥2 days from the highest previously recorded Glasgow coma score (including baseline) or the occurrence of any of the following adverse events: cerebellar symptoms, coma, hemiplegia, paraplegia, seizures, cerebral herniation, new onset blindness or deafness, or cranial nerve palsy.

***Longitudinal measurements of intracranial pressure during the first 2 weeks.*** Intracranial pressure (ICP) will be measured at study entry, day 3, 7, and 14, and if clinically indicated (depending on local practice). The main outcomes are longitudinal ICP-measurements until day 14.

Clinical need and local practice will determine the frequency of lumbar punctures post day 14 and clinician’s diagnoses of raised ICP based upon the presence of headache, nausea, diurnal and postural variation, relief with lumbar puncture and presence of papilloedema. Thus, these measures will only be descriptively analyzed.

***CD4 count at 10 weeks.*** CD4 count measurement is indicated in HIV infected patients, and CD4 lymphopenia has been described in HIV uninfected patients with cryptococcal meningitis. Moreover, Tamoxifen may reduce CD4 cell apoptosis which may be beneficial ^47^.

***Blood and CSF concentrations of amphotericin, Tamoxifen and fluconazole.*** Pharmacological data on antifungal drugs are lacking. Dosing has been inferred from studies in healthy volunteers, or patients with other diseases. There are no studies of combination therapy at the doses currently recommended ^48– 50^, and no data on the effect of Tamoxifen on fluconazole concentrations. This study is an opportunity to improve our understanding of the pharmacokinetics and pharmacodynamics of the currently recommended treatment combination in patients in a real world setting. It offers the chance to better understand how plasma concentrations of the drugs relate to clinical and microbiological outcomes, and therefore how treatment regimens might be improved. All patients will undergo pharmacokinetic sampling to enable the description of the concentrations of Tamoxifen and fluconazole in plasma and CSF, and of amphotericin in blood, and relate these to the rate of clearance of yeast from CSF. The yeast burden at each lumbar puncture will be determined using standard methods as in previous OUCRU studies ^9^. PK and PD data will eventually be considered alongside data from the previously published CryptoDex trial ^9^.

***Pathogen and host gene expression.*** The second aim of the study is to improve understanding of the pathogenesis of CM by analyzing gene expression of both the pathogen and host. Fungi require a minimum set of interacting properties for pathogenicity ^51– 53^. For *Cryptococcus*, it is clear that pathogenicity factors identified to date represent only a fraction of the genes vital to cause disease ^52^. Data suggest that variation in gene expression may be a major virulence determinant ^52– 55^. In Vietnam where we have identified 14 circulating lineages of *C. neoformans*, and found that lineage is associated with disease phenotype ^56^. In particular, we have identified a clade (VNIγ) responsible for almost all (>92%) of cases in apparently immunocompetent patients. We hypothesize that this strain has increased pathogenicity. These VNIγ strains also account for 35% of disease in HIV-infected patients. Whole genome sequencing of Vietnamese isolates has demonstrated substantial differences between VNIγ and the most closely related clade, including over 100 gene insertions and deletions, and multiple single nucleotide polymorphisms (≈40k) ^57^. To better understand exactly which genes define disease in HIV infected and uninfected patients, we will compare their relative expression according to infecting sequence type and host immune phenotype. Drainage of CSF to manage raised CSF pressure (see extended data appendix 1 ^58^) is a key part of the usual management of cryptococcal meningitis, and together with the high burden of yeasts in CSF in cryptococcal disease, makes the measurement of gene expression during disease using RNAseq feasible. Whenever a patient undergoes lumbar puncture, the CSF opening pressure will be measured and if elevated CSF will be drained according to international guidelines ^6^. These state that the volume of CSF drained should be sufficient to reduce the pressure to half the opening pressure or to within the normal range (<18cm CSF) - whichever comes sooner. Typical volumes of CSF drained are 10–40ml. Following removal, CSF will be aliquoted for any indicated standard analyses (eg glucose, quantitative count, PK studies), and the remainder will undergo immediate processing for RNA extraction.

RNAseq is not organism specific and will not distinguish between pathogen and host (human) gene expression – all genes will be captured. However, yeast cells will be dominant in CSF and the majority of genes measured will be from *Cryptococcus*. For patients who do not consent to having their own gene expression measured, only yeast gene expression will be analysed - the human signal will be subtracted. However, our past experience suggests that almost all patients are comfortable to take part in human genetic studies following explanation of the purpose of the study.

## Design

### Study design

**Figure 1.**
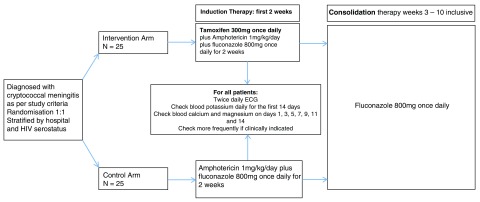
Trial design and flow diagram.

A randomized, open-label trial with 2 parallel arms of standard antifungal therapy versus Tamoxifen augmented antifungal therapy during the first 2 weeks (induction phase) of treatment (see Figure 1). The study will recruit patients in two sites in Ho Chi Minh City, Viet Nam: the Hospital for Tropical Diseases (HTD), and the Department of Tropical Medicine, Cho Ray Hospital (CRH). The study is pragmatic, designed to maintain relevance through trialing Tamoxifen in the context of the best standard of locally available care. 25 patients will be enrolled into the two study arms (intervention versus control). All anti-fungal administration will be directly observed by ward staff.

### Intervention arm: Induction phase treatment (days 1–14)

Tamoxifen will be given orally in a dose of 300mg/day for the first 14 days following randomization. It will be administered by nasogastric tube where patients are unconscious. In addition patients will receive amphotericin 1mg/kg once daily i.v. and fluconazole 800mg once daily orally. The Tamoxifen will be administered in the morning combined with amphotericin and fluconazole dose.

### Control arm: Induction phase treatment (days 1–14)

Patients will receive amphotericin 1mg/kg/day i.v. combined with fluconazole 800mg once daily orally for the first 2 weeks. Amphotericin and fluconazole will be administered simultaneously.

### Continuation phase of treatment (days 15–70)

After the first 2 weeks of study treatment, all patients will receive fluconazole 800mg/day for 8 further weeks, until the study end. At this point, HIV infected patients will be switched to long term secondary prophylaxis with fluconazole 200mg/day as per standard practice. For HIV uninfected patients, the decision to continue antifungal treatment, and at which dose, will be made on a case by case basis by the attending physician in consultation with the patient.

### Study Population

All HIV infected and uninfected adult patients with a diagnosis of cryptococcal meningitis presenting to the study centres will be eligible to enter the study, subject to meeting the inclusion/exclusion criteria.

### Trial Location

The study will recruit patients at sites on Ward E, Ward C and the Viet-Anh Ward, at HTD and in the Department of Tropical Medicine at CRH.

### Inclusion criteria

- Age ≥ 18 years
- Cryptococcal meningitis defined as a syndrome consistent with CM and one or more of:
  ○ positive CSF India ink (budding encapsulated yeasts),
  ○ *C. neoformans* cultured from CSF or blood,
  ○ positive cryptococcal antigen Lateral Flow Antigen Test (LFA) in CSF

- Informed consent to participate given by patient or acceptable representative
- Known HIV infection status, or patient agrees to HIV testing on this admission

### Exclusion criteria

- Pregnancy or breast-feeding
- History of thromboembolic disease such as pulmonary embolism or deep venous thrombosis
- On anti-coagulant medication
- On medication known to prolong the QT interval other than fluconazole, such as fluoroquinolones or antidepressants.
- Known cardiac conduction defect including long QT syndromes
- QTc at baseline > 500ms
- Currently receiving treatment for cryptococcal meningitis and having received ≥4 days of anti-cryptococcal meningitis therapy
- Known allergy to Tamoxifen
- Currently or history of receiving treatment with Tamoxifen for breast cancer or other indication
- Current or history of uterine cancer including endometrial cancer and uterine sarcoma
- Renal failure (defined as creatinine>3*ULN, despite adequate hydration)
- Failure to consent – the patient, or if they are incapacitated, their responsible relative, declines to enter the study
- Allergy to amphotericin B or fluconazole

## Study procedures

### Recruitment

The target population for study screening is any patient known or suspected to have cryptococcal meningitis. To enter the study patients must be confirmed to have cryptococcal meningitis according to the definition in the inclusion criteria. According to the clinical care of the treating hospital, patients suspected of having cryptococcal meningitis will undergo:

a) an IMMY lateral flow cryptococcal antigen test (LFA) on serum, whole blood, plasma, or urine

AND/OR

b) blood culture for *Cryptococcus*

AND/OR

 c) a lumbar puncture with an lateral flow cryptococcal antigen test (Ref CR2003, IMMY, OK, USA), and/or microscopic examination of CSF, and/or culture of CSF. 

When the results of the IMMY lateral flow test (plasma, serum, urine or CSF), or CSF microscopy, or blood or CSF culture are available study staff may approach the patient regarding the study. Only patients 18 years or older who are not known to be pregnant and who have evidence of cryptococcal disease from one of the specified tests will be approached. 

A study staff member will invite the patient to discuss the details of the study. If the patient is judged by the staff to be unfit or unable to give informed consent, an acceptable representative will act on their behalf for the following procedures. The study staff will give the patient/representative a copy of the informed consent form and explain the details of the study including the study procedures, risks and benefits, financial and confidentiality considerations, treatment alternatives and how to obtain more information. The study staff will invite the patient/representative to ask questions and will endeavour to ensure that s/he understands the information given. The study staff will then ask the patient/representative to consider study participation. Those who refuse consent will be treated as per the best available standard care and will not have any study related procedures performed. 

Those who consent to the study will sign and date two copies of the informed consent form. The study staff will also sign and date the two copies. 

If the patient/representative is illiterate, a witness who is not a member of the study staff will be present during the informed consent discussion. The informed consent form will be read to the patient/representative in the presence of the witness. If the patient/representative agrees to participate, the form will be signed and dated by the witness. Consented patients will be screened for eligibility.

### Screening

Only patients who are 18 years or older, who are not known to be pregnant, and who have at least one of positive CSF or blood/serum/plasma/urine LFA test, positive blood or CSF culture, or positive CSF microscopy will be consented. Consented patients will undergo the following screening procedures/tests. In the case of an unconscious patient, information will be obtained from a knowledgeable relative or caregiver.

- Medical history will be taken including: 1) signs and symptoms consistent with cryptococcal meningitis, 2) allergy to Tamoxifen, 3) history of breast or uterine cancer, venous thromboembolic events and > 4 days anti-fungal therapy.

- All females of child bearing age will have a urine or blood pregnancy test.

- 12 lead ECG to exclude cardiac conduction defects

- Creatinine level.

- HIV status will be confirmed from clinical history or testing as per standard of care. HIV testing is indicated and part of routine care for all patients with cryptococcal meningitis, since it defines the need for antiretroviral therapy and prophylaxis against other opportunistic infections.

- A lumbar puncture will be performed on all patients to obtain CSF. The opening CSF pressure will be determined using a spinal manometer. CSF will be tested by: 1) India ink stain or equivalent, 2) quantitative culture, 3) lateral flow cryptococcal antigen test. Cultured isolates will be stored for subsequent studies. Residual CSF will be stored for cryptococcal gene expression studies which will be done in patients who pass screening and enter the full study.

- If a lumbar puncture was done recently (within 48 hours) for clinical care and volume of fresh CSF remains available for these tests (stored according to established SOPs), the lumbar puncture need not be repeated.
- If the patient underwent a recent lumbar puncture, an additional puncture will be performed (provided there are no contra-indications) if any of the following are true: 1) there is uncertainty regarding the microbiological diagnosis, 2) raised intracranial pressure is suspected, 3) the previous puncture was >2 days prior and no effective treatment has been given, 4) it is required for standard care. If none of the above are true or if the patient refuses further lumbar puncture, they may be randomized without additional lumbar puncture provided they are eligible for the study.
- If the lumbar puncture was not recent, it will be repeated (provided there are no contraindications) in order to confirm the diagnosis, and to determine CSF pressure and fungal burden.

All lumbar punctures require verbal or written consent according to local standard clinical practice. Failure to consent is a contraindication to the procedure.

When all inclusion and exclusion criteria are verified, eligible patients will be randomized to treatment.

**Figure 2.**
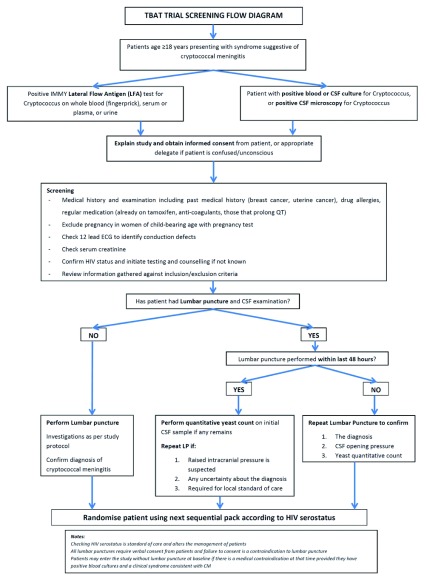
Estimated power based on the two simulation studies.

Patients who are determined to be ineligible will be withdrawn from the study and the reason recorded. Patients withdrawn from the study will be treated according to the best available standard care. Screening flow is illustrated in Figure 2.

The number of patients who do meet inclusion/exclusion criteria, but are not enrolled to the study will be recorded.

### Randomization

Randomization will be 1:1 to either Tamoxifen augmented or standard antifungal therapy. Block randomization with stratification by hospital of enrolment and by HIV infection status, and variable block sizes of 4 and 6, respectively, will be used to assign subjects to treatment. The randomization list will be generated according to OUCRU standard operating procedures. In brief, the Research Biostatistician will set up statistical code to generate the randomization list and transfer it to the central Study Pharmacist. The Study Pharmacist will change the random seed, i.e. the initialization of the random numbers generator, in the statistical code in order to blind the Research Biostatistician and then run the code to prepare the final randomization list for treatment preparation. The randomization list will be password protected and stored on a secure server to which only the Study Pharmacist has access.

Based on the randomization list, the central Study Pharmacist will generate identical sealed treatment packs for each study ID and distribute them to the sites in batches as required. All treatment packs will be identical in appearance and will contain either Tamoxifen and fluconazole or fluconazole alone. Each pack will contain sufficient fluconazole for the first 2 weeks of treatment. Enrolment logs specific to each ward will be used to assign patients to the next available sequential number and corresponding sealed treatment pack.

## Patient management

### Initial evaluation

On admission all patients will have a full clinical assessment and examination.

Study entry laboratory tests will be performed as per the study schedule in Table 2.

**Table 2.** Trial Assessment and Investigation Schedule *.

Action	Day 1(study Entry)	Day 3	Day 5	Day 7	Day 9	Day 11	Day 14	Day 21	Day28	Day 42	Day 70
**Take informed consent**	X										
Clinical Assessment **	X	X		X		X	X	X	X	X	X
**Full blood count**	X			X							X
**Sodium, Urea, Creatinine,** **Glucose**	X	X		X		X	X	X	X	X	X
**Magnesium and Calcium**	X	X	X	X	X	X	X	X			
**ALT and AST**	X			X			X				
**Potassium**	Daily for first 14 days							X	X		
**CD4 count**	In first week										X
**HIV test**	X										
**Blood cultures**	X										
**Cerebrospinal fluid (CSF)** **opening pressure**	X	X		X			X	if clinically indicated			
**Cryptococcal Lateral flow** **antigen test (CSF)**	X										
**CSF Gram's stain, India ink**	X	X		X			X				
**CSF cell count, protein,** **glucose**	X	X		X			X				
**CSF TB smear/geneXpert**	During first week										
**CSF Yeast quantitative** **culture**	X	X		X			X				
**Store *C. neoformans*** **isolate**	X	X		X			X				
**CSF for Pharmacokinetics**	X	X		X							
**Plasma for** **pharmacokinetics**	X			X							
Sputum TB smear ***	X										
**Electrocardiogram**	Twice daily for first 14 days										
**Chest X-ray**	X										
**Approximate total blood** **volume mL**	21.5										
**Approximate total CSF** **volume mL**	up to 25	up to 25		up to 25			up to 25	up to 25	up to 25	up to 25	up to 25

* Study drug is given daily from day 1 – day 14** GCS Assessment is daily while an in-patient. When outpatient, assessment can take place at the scheduled time + up to 5 days (e.g. 4 week assessment on day 28-33). ***at doctor’s discretion **** volume determined by CSF pressure N.B.: Blood volumes are estimates

**Table 3.** simple visual assessment.

Record the best performance of each eye	
Function	Score
Normal	1
Blurred	2
Finger counting	3
Movement perception	4
Light perception	5
No light perception	6

A baseline chest radiograph will be performed. A CT or MRI brain scan will be performed if there is evidence of raised intracranial pressure or focal neurological abnormalities according to local practice and resources.

### Hospital admission

Intravenous amphotericin B is administered for 14 days necessitating hospital admission during this period. Study treatment and antifungals will be directly administered during this time period.

### Clinical monitoring

Patients will have daily Glasgow Coma Score and review as per standard care until discharge from hospital. The decision to discharge patients from the hospital is at the attending physician’s discretion and is based upon the clinical status of the patient. Following discharge patients will be seen weekly until 4 weeks, at 6 weeks and at 10 weeks. If the exact visit day is not feasible, scheduled visits can occur at up to 5 days following the stipulated time to account for weekends and holidays (for example, the 4 week review should occur at on day 28–33 following randomisation, week 10 visit on day 70–75). Patients will be monitored closely for

- Death - the date of death and cause will be recorded
- New neurological events (onset of new focal neurological signs or fall in Glasgow coma score of ≥ 2 points for ≥ 2 days, following > 7 days clinical stability or improvement after randomization)
- Drug-related adverse events
- New or recurrent AIDS defining illnesses
- Visual deficit (at week 10)
- IRIS
- Raised intracranial pressure (clinical or measured)

Uniform management of patients and recording of data will be ensured by the local study staff who will do clinical assessments daily while admitted and at follow-up visits. Uniform management and data recording will be ensured through training of study staff, standard operating procedures for the administration of drugs and identification and management of specific complications, and the use of a clinical reporting form that has been developed in previous OUCRU studies. The study has a follow-up period of 10 weeks post-randomisation, and the full study analysis will be performed and the data submitted for publication when the last patient under follow-up has reached this timepoint.

### Adherence monitoring

All administration of study and antifungal drugs will be directly observed and recorded in the treatment medication sheet of the CRF during the in-patient stay. On discharge, sufficient fluconazole will be dispensed to meet the patient’s needs until 10 weeks following randomization, plus 3 extra days. At each study visit a pill count will be performed to monitor adherence to antifungal therapy.

### Cardiac monitoring

ECGs (50mm/sec) will be performed twice daily immediately before and 2 hours after administration of Tamoxifen during the first 14 days, and on days 21 and 28. ECGs can be repeated more frequently according to the patient’s needs. The QTc will be calculated as described in section ’Prolongation of the QT Interval’.

*Laboratory monitoring*

## Blood

Inpatient laboratory monitoring will be as shown in the study schedule ( Table 2). Patients will have daily monitoring of potassium and alternate day monitoring of creatinine, calcium and magnesium levels for the first 2 weeks of the study(i.e. days 1, 3, 5, 7, 9, 11, and 14. Following this point levels will be monitored as clinically indicated. Full blood count will be measured at study entry, and at days 7 and 14, and thereafter as clinically indicated. CD4 count will be measured in all patients during week 1 and at day 70.

## Cerebrospinal fluid

Lumbar puncture will be performed on days 1, 3, 7 and 14 following randomization. CSF opening and closing pressure will be measured on each sampling. Following the initial diagnostic lumbar puncture, routine diagnostic investigations will be performed according to clinical need. Whenever CSF is sampled, the fungal burden will be determined by culture (0.5mL), an aliquot immediately processed and frozen to -80°C for subsequent drug level measurement (1-1.5mL), and the remainder of any CSF (ie above that needed for clinical care and the PK measurements, and between 1-40mls, depending upon the amount needed to be removed to manage the CSF pressure) immediately processed for RNA extraction for gene expression studies. Rarely (approximately 0.5%) TB meningitis can occur at the same time as cryptococcal meningitis. Patients should have CSF tested by GeneXpert or smear or other nucleic acid amplification test during the first week of study treatment.

## PK sampling

### Plasma sampling for PK

Participation in the plasma PK study will require additional consent from the patient. Patients who agree to participate will have baseline bloods taken prior to medication being given, but amphotericin will be prepared, ready for administration, so that the infusion can commence without delay following venesection. 1.5ml of blood will be taken in a plain tube and transported in iced water to the laboratory where it will be immediately centrifuged and the plasma frozen to -80°C for storage. This will be the 0hr sample. The standard 4 hour amphotericin infusion will then commence, and oral fluconazole and Tamoxifen will be administered. Patients will be sampled for PK a total of 5 times each on day 1 and day 7. The total volume of blood drawn will be 7.5mL on each day. The exact timing of blood draws is currently being modelled based on previous Vietnamese data by Prof Hope in Liverpool, such that the most efficient sampling schedule is used. The timing of administration of all antifungal drugs will be meticulously recorded. In addition, every time a lumbar puncture is performed, a paired plasma sample will be taken (1.5mL).

### CSF sampling for PK

PK CSF sampling will not require additional lumbar punctures (see section ’Clinical Monitoring’, subsection ‘Cerebrospinal fluid’). Rather, the timing of all lumbar punctures will be meticulously recorded along with the timing of antifungal drug administration. 1-1.5ml of CSF will be required for PK studies. PK studies will only be done if there is sufficient sample available after routine clinically indicated investigations have been performed. Every time a lumbar puncture is performed, a paired plasma sample (1.5mL) will be taken for drug concentration determination (see section ‘Laboratory Monitoring’, subsection ‘PK Sampling’).

## Other investigations

Other investigations may be performed as clinically indicated. Data for the following will be recorded when performed for routine clinical care:

- CSF, if neurological deterioration (Gram stain and routine culture, ZN stain and mycobacterial culture, India ink stain and fungal quantitative culture)
- Sputum, if symptomatic (routine culture, ZN stain)
- Urine culture, if urinary symptoms (urine culture)
- Stool culture, if prolonged diarrhoea (microscopy, culture and parasites)
- Blood cultures, if persistent fever
- Lymph node aspiration (routine and mycobacterial cultures)
- Blood glucose will be measured when CSF is examined or if hyper or hypoglycaemia is suspected
- Liver function tests

A window period of +/- 2 days outside of the scheduled laboratory tests will apply to all tests except for electrolytes including potassium, magnesium and calcium, and those needed at baseline for inclusion/exclusion criteria.

### Evaluation of Cryptococcus isolates

All *Cryptococcus* isolates will be speciated using classical mycological and molecular methods, and typed through genome sequencing and phenotypic testing. All isolates will undergo susceptibility testing for antifungal drugs and Tamoxifen. Isolates will be securely stored in OUCRU, HTD.

### Imaging

A chest X-ray will be performed on study entry if it has not been done at the time of diagnosis. The result will be recorded in the CRF. Brain imaging is not mandated by the study. The decision to perform brain imaging will be according to local practice. Results of brain imaging when available will be recorded. The brain imaging modality of choice will be MRI.

*Other treatment*

## Antifungal treatment

All patients – control and intervention arm - will receive antifungal treatment consisting of 2 weeks of intravenous amphotericin B 1mg/kg/day combined with high dose fluconazole (800mg once daily), followed by fluconazole 800mg once daily for 8 weeks (10 weeks in total). This is locally feasible and consistent with recent guidelines ^6, 7^. Amphotericin will be administered according to recommended guidelines (see extended data, appendix 2 ^58^). Following the 10 week period of therapy, HIV infected patients, provided they have responded to treatment, will receive long term secondary prophylaxis with fluconazole 200mg/day as per standard of care ^7^. For HIV uninfected patients, the dose of antifungal therapy after 10 weeks will be determined on a case by case basis depending on response to treatment and the attending physician’s judgement regarding the likelihood of there being some other chronic immunosuppressive condition. After the initial 2 weeks of the study, modification of antifungal therapy for any patient can be made according to the patient’s needs and the judgment of the attending physician. Any changes to antifungal therapy will be recorded in the case record form. The cost of antifungal treatment until 10 weeks following randomization will be covered by existing local financial support or trial finances. Following the final follow-up visit patients will continue in care under the existing local services.

## Antiretroviral therapy

All HIV infected patients will be referred to local HIV services as soon as practicable, preferably while still admitted to hospital, to ensure that they have access to locally available HIV services including counseling and ARVs.

The recent COAT trial suggests that early (within 5 weeks of diagnosis and institution of effective antifungal therapy) introduction of ARVs is associated with worse outcomes. Therefore the study protocol recommends that ARVs are begun at least 5 weeks after institution of antifungal treatment, provided the patient has made a good response. The date that ARVs are started (or stopped) in patients in the study will be recorded. Where a patient is already receiving ARVs at the time of diagnosis, the decision to continue them will depend upon the decision of the local attending physician who will make a judgement regarding whether the cryptococcal meningitis represents antiretroviral failure or not.

## Opportunistic infection (OI) prophylaxis for immunosuppressed patients

HIV infected patients will be profoundly immunosuppressed and will receive prophylactic therapy against pneumocystis pneumonia with daily trimethoprim-sulfamethoxazole, in accordance with local guidelines and practices. If other patients with non-HIV related immunosuppression are identified, then they will receive OI prophylaxis according to the local physicians’ judgement.

### Data on concomitant medications

At each visit, information on other medications, including start dates and reason for taking them, will be documented in the case record forms. 

## Management and prevention of potential cardiac adverse events

Possible side effects of Tamoxifen are described earlier.

### Electrolyte disturbances and raised creatinine

Electrolyte disturbances are common in cryptococcal disease, usually precipitated by amphotericin treatment. The likelihood of such disturbances are mitigated by pre-emptive electrolyte replacement, sodium loading, and ensuring adequate hydration. It is routine practice to administer 2 litres of normal saline and 80mmol/L of potassium chloride daily to patients receiving amphotericin treatment. All patients in the study will receive such pre-emptive fluid and electrolyte replacement unless they are fluid overloaded or have elevated serum potassium concentrations. In the event of the serum creatinine being greater than 300umol/L, the next amphotericin dose will be missed, and the hydration status of the patient re-assessed and corrected. Amphotericin will be restarted when the serum creatinine is <200umol/L.

### Prolonged QTc

Prolongation of the QT interval will be actively monitored with daily pre and 2 hour post Tamoxifen dose 12 lead ECGs at 50mm/s for the first 2 weeks of treatment, and weekly after that, unless they are indicated more frequently. Trained study staff will measure and calculate the corrected QT interval. It is expected that around 10–50% of patients will develop prolonged QTc intervals while on treatment ^13, 16^. With some drugs, a prolonged QTc can lead to a polymorphic ventricular tachyarrhythmia (Torsades de Pointes, TdP) which can be life threatening. However, this has not been described in patients receiving Tamoxifen, including in those receiving higher doses as will be used in this study ^12, 13, 16, 26^. Prolongation of the QT interval has also been reported in patients receiving fluconazole, but TdP has only rarely been described with less than 10 reports published at the time of writing, despite its extensive use ^38, 40^. Moreover, in a case series of cancer patients receiving fluoroquinolones (which also prolong the QT interval) combined with fluconazole, despite QT prolongation there were no episodes of arrhythmias ^42^. In this study QT prolongation will be managed in line with American Heart Association guidelines as described below ^32^. Any risk of TdP will be mitigated by close monitoring and management of electrolytes, particularly potassium, calcium and magnesium. The use of other drugs which can prolong the QTc and have been associated with TdP, other than fluconazole, will be prohibited during the first 14 days following randomization. Appendix 4 of Extended data ^58^ lists such drugs.

If the QTc is >500ms,

1. Do not administer further tamoxifen until the QTc is ≤ 500ms.
2. Immediately recheck blood potassium, calcium and magnesium levels.
3. Complete a Cardiac Adverse Event form from the CRF
4. Correct abnormal electrolytes into the mid normal range with intravenous supplementation (potassium chloride, calcium gluconate or chloride, and magnesium sulfate). Use intravenous access separate to the amphotericin infusion when administering electrolytes.
5. Repeat the ECG, a 30 second rhythm strip, and serum electrolytes 2 hours prior to the next due dose of Tamoxifen.
  a) If the QTc is >500ms, the due dose of tamoxifen should be omitted.
  b) If the QTc is ≤500ms but there are extra-systoles, the next dose of tamoxifen should be omitted
  c) If the QTc is ≤500ms and there are grade 2 or greater abnormalities in serum potassium, magnesium or calcium levels, the next dose of tamoxifen should be omitted.
  d) If the QTc is ≤500ms, and there are no extra-sytoles, and the calcium, potassium and magnesium levels are normal or no more than grade 1 abnormal, the next due dose of tamoxifen can be given.
6. Where the QTC is >500msec, the administration of tamoxifen is suspended and the patient must
  a) have daily ECGs and monitoring and correction of electrolytes until the QTc is less than 500ms.
  b) When the QTc is <500ms, Tamoxifen can be restarted according to the guidance in 5 a-d above.
7. Tamoxifen is administered until day 15 after randomization. All due, received and missed drug doses must be recorded in the study case record form (CRF).
8. Any patient where the QTc is greater than 500ms AND is accompanied by syncope OR arrhythmia should have review by a cardiologist.

### Ventricular tachyarrhythmia

In the event of TdP or any other ventricular tachyarrhythmia, patients will receive oxygen and be transferred to a high care environment for continuous cardiac monitoring. If there is cardiovascular compromise, resuscitation will follow normal practice. Electrolytes will be rechecked, corrected if needed, and administration of Tamoxifen and fluconazole suspended.

TdP usually occurs in unsustained bursts that spontaneously terminate. However, **the specific treatment of TdP is intravenous magnesium**, given as Magnesium sulfate in a dose of 1–2 g intravenously over 5 to 10 minutes diluted in 50–100mls of 5% dextrose. This can be repeated in 5–15 minutes. Alternatively, a continuous infusion can be started at a rate of 3–10 mg/min. Note: Magnesium is effective even in patients with normal magnesium levels.

**Temporary transvenous pacing** can be used to treat refractory TdP. Temporary pacing is used based on the fact that the QT interval shortens with a faster heart rate. Therefore, pacing can be effective in terminating torsade. Atrial pacing is the preferred mode because it preserves the atrial contribution to ventricular filling and also results in a narrower QRS complex and hence a shorter QT. Pacing should be instituted at a rate of 90–110 bpm until the QT interval is normalized. Overdrive pacing may be necessary at a rate of up to 140 bpm to control the rhythm.

**Patients who are in extremis** with TdP, or who develop ventricular fibrillation, should be treated with **electrical cardioversion or defibrillation.**

All arrhythmias are adverse events and must be recorded on the appropriate form in the CRF.

Patients who have arrhythmias should be reviewed by a cardiologist.

Fluconazole should be restarted when the arrhythmia is resolved for 24 hours. Tamoxifen will not be restarted for any patient who has a ventricular tachyarrhythmia.

### Syncope

Any patient who has sudden onset of loss of consciousness will be assessed by a clinician according to normal practice. ECG and electrolytes will be checked and corrected. The patient will be monitored in a high care environment. Tamoxifen administration will be stopped and not restarted. Patients who have syncope due to suspected arrhythmia should be reviewed by a cardiologist.

## Stopping study drug

Occasionally it can be necessary to stop the study intervention (Tamoxifen). Stopping a patient’s treatment does not mean that they have withdrawn from the study. Patients continue in the study and follow the protocol schedule for visits and investigations until its conclusion.

### Indications for stopping Tamoxifen

Tamoxifen is generally a well tolerated drug. Studies from breast cancer suggest increased risks of pulmonary embolism, and uterine malignancies, but the absolute risks of these are very low and they are associated with prolonged use over many months rather than short course duration, as will be used in this study ^29^. It is unlikely that the 2 weeks duration of treatment in this study will increase these risks. Prolongation of the QTc interval is to be expected in this study and does not necessarily indicate a risk of ventricular arrhythmia. The management of study drug in the face of prolonged QTc (>500ms) has been described in section ‘Management of Adverse Events’, subsection ‘Prolonged QTc’. However, Tamoxifen will be stopped and not restarted if any of the following events occur in a particular patient:

1. deep venous thrombosis
2. venous thromboembolism
3. syncope
4. ventricular tachyarrhythmia

## Withdrawal from the trial

Patients may voluntarily withdraw from the trial for any reason. If this occurs, the patient will be referred to standard clinical care facilities. The patient’s withdrawal from the trial will not affect their access to the best standard of care within the local health system. With the agreement of the patient, clinical and laboratory assessment should be performed and recorded at the time of withdrawal. Patients may also decide to stop study treatment without withdrawing from the study, in which case Tamoxifen will be stopped and follow-up will continue as per the study schedule.

## Adverse events

### Definition of adverse events

An adverse event (AE) is any undesirable event that occurs to a study participant during the course of the study whether or not that event is considered related to the study drug. An AE can, therefore, be any unfavorable and unintended sign (including an abnormal laboratory finding, for example), symptom, or disease temporally associated with the study drug, whether or not considered related to the study drug.

Examples include:

- An increase in severity (grade) or frequency of a pre-existing abnormality or disorder (events that are marked by a change from the participant’s baseline/entry status, including changes in blood tests such as electrolytes)
- All reactions from sensitivity or toxicity to study drug
- Injuries or accidents (e.g., for a fall secondary to dizziness, “dizziness” is the event and the injury secondary to the fall is the “outcome”)
- New clinically significant abnormalities in clinical laboratory values, physiological testing or physical examination.

Stable chronic conditions, such as arthritis, which are present prior to clinical trial entry and do not worsen are not considered AEs and will be documented in the subject’s clinical chart as medical history. 

Clinical or laboratory events are considered adverse events only if they occur after the first dose of study treatment and before the patient completes trial participation. (See below for reporting of adverse events.)

### Definition of Serious Adverse Events

An AE is considered to be "serious" if it results in one of the following outcomes

- Death,
- Life-threatening event (the subject was at immediate risk of death at the time of the event; it does not refer to an event which hypothetically might have caused death if it were more severe),
- Inpatient hospitalization or prolongation of existing hospitalization
- Persistent or significant disability/incapacity (a substantial disruption of a person's ability to conduct normal life functions),
- Congenital anomaly/birth defect
- Important medical event that may not be immediately life-threatening or result in death or hospitalization but may jeopardize the patient or may require intervention to prevent one of the other outcomes listed in the definition above.

### Definition of Unexpected Serious Adverse Events

Unexpected Serious Adverse Events are untoward medical events which fit one or more of the criteria for SAEs above and which are not considered a part of normal clinical progression of disease or an expected drug reaction. Any event which becomes of concern to the investigators or study doctors during the course of the trial may be reported as a USAE.

### Assessment of Adverse Events

Adverse events will be defined according to the Common Terminology Criteria for Adverse Events (CTCAE). New AIDS defining events will be defined according to the revised CDC criteria modified for this trial (see extended data, Appendix 3 ^58^). In the event that an adverse event is not described within the CTCAE definitions, or is a new AIDS defining event, the following generic severity grading will be used: 

**Grade 1 Mild;** asymptomatic or mild symptoms; clinical or diagnostic observations only; intervention not indicated.

**Grade 2 Moderate;** minimal, local or noninvasive intervention indicated; limiting age-appropriate instrumental Activities of Daily Living (ADL)*.

**Grade 3 Severe or medically significant but not immediately life-threatening;** hospitalization or prolongation of hospitalization indicated; disabling; limiting self-care ADL**.

**Grade 4 Life-threatening consequences**; urgent intervention indicated.

*Instrumental ADL refer to preparing meals, shopping for groceries or clothes, using the telephone, managing money, etc.

**Self-care ADL refer to bathing, dressing and undressing, feeding self, using the toilet, taking medications, and not bedridden.

 [Note: “Life-threatening” as a severity grade is not necessarily the same as “life-threatening” as a “serious” criterion used to define a serious adverse event. The former is a “potential” threat to life and the latter is an “immediate” threat to life.]

A **laboratory abnormality** only needs to be recorded as a clinical adverse event if it is associated with an intervention. Intervention includes, but is not limited to, discontinuation of a current treatment, dose reduction/delay of a current treatment, or initiation of a specific treatment. In addition, any medically important laboratory abnormality may be reported as an adverse event at the discretion of the investigator. This would include a laboratory result for which there is no intervention but the abnormal value suggests a disease or organ toxicity. Laboratory events will be graded according to CTCAE definitions.

If clinical sequelae are associated with a laboratory abnormality, the diagnosis or medical condition should be reported as the adverse event (e.g., renal failure, haematuria, cardiac arrhythmia) - not the laboratory abnormality (e.g., elevated creatinine, urine RBC increase).

## Recording and reporting of Adverse Events and protocol violations

### Adverse event recording

Grade 3 and 4 adverse events will be **recorded** in the case report form and entered to the study database. Grade 1 & 2 events will not be recorded since in a severe disease such as cryptococcal meningitis the number of low grade adverse events is likely to be high representing a large reporting burden which may impact upon the quality of recording and reporting of more important grade 3 and 4 adverse events.

### Adverse event reporting

Serious adverse events and serious unexpected adverse events will be **reported** to the Principal Investigator within 3 days of occurrence, or sooner according to local requirements. The Principal Investigator will report all unexpected serious adverse events to the DSMB within 10 days of occurrence. Unexpected serious adverse events will be reported to the responsible ethical committees within 10 days of occurrence or as required by the committee. Serious adverse events will be reported to the local IRBs on a monthly basis.

### Protocol violations

Protocol violations are events which contradict or omit protocol instructions. Violations which compromise patient safety or the integrity of trial data will be recorded and reported to the responsible Ethics Committees as required by the regulations of each Committee.

## Statistics

### Sample size and power considerations

Sample size considerations were based on simulation using data from two previously published trials in cryptococcal meningitis conducted in Vietnam ^8, 9^: 96 patients from the BK trial (all recruited at HTD) with at least one quantitative fungal count measurement randomized to amphotericin B plus fluconazole, and 97 randomized to amphotericin B plus flucytosine; plus 49 patients from the CryptoDex trial recruited at HTD or Cho Ray Hospital randomized to placebo (all of these received antifungal treatment with amphotericin B plus fluconazole).

The first set of simulations assumed that the Tamoxifen group was associated with a similar effect on Early Fungicidal Activity (EFA) as amphotericin B plus flucytosine. For sample sizes ranging from 15-30 patients per group, we repeatedly sampled patients from both included BK study arms of that size with replacement, analyzed them, i.e. compared EFA between the two groups, and then determined the proportion of significant results (i.e. the estimated power).

For the second set of simulations, the control group consisted of the combined 145 patients from the BK study and 04CN receiving amphotericin B plus fluconazole. For assumed EFA treatment effects ranging from -0.16 to -0.10 log _10_CFU/ml per day faster declines in the intervention group, we generated hypothetical intervention group data by simulating this treatment effect on top of the observed fungal count measurements for patients receiving amphotericin B plus fluconazole. Power was then determined based on resampling-based simulation as for the first set of simulations.

Estimated power was based on 10 000 repetitions of each simulation experiment. Estimating EFA based on a mixed model accounting for the detection limit of fungal count measurements was not feasible because this would require excessive computing time. Thus, we used a simple mixed model ignoring the detection limit for the simulations instead but removed values after the first undetectable (zero) fungal count measurements and kept the first fungal count of zero only if it lead to a steeper estimated least-squares decline for that patient compared to omitting the measurement.

**Figure 3.**
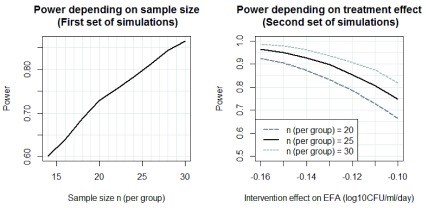
Screening study flow diagram.

Results for both sets of simulations are shown in Figure 3. Based on the first simulation study, n=25 subjects per group provide approximately 80% power to detect a difference in EFA of similar size as the difference between amphotericin B plus fluconazole and amphotericin B plus flucytosine observed in the BK study. Based on the second simulation study, n=25 subjects per group provide 80% and 90% power to detect a difference in EFA of -0.11 or -0.13 log10CFU/ml/day, respectively. We consider that this size of effect is likely to deliver a clinical benefit. All simulations were performed using R software version 3.2.2 ^59^.

*Analysis*

## Analysis of the primary endpoint: early fungicidal activity (EFA)

All recorded longitudinal quantitative fungal count measurements up to day 17 (allowing for some delays in the day 14 measurements) will be included in the analysis. EFA will be modeled based on a linear mixed effects model with longtitudinal log _10_-CSF quantitative culture fungal counts as the outcome, interaction terms between the treatment groups and the time since enrolment of the measurement as fixed covariates and random patient-specific intercepts and slopes. The lowest measurable quantitative count is 5 CFU/ml and values below the detection limit (which correspond to recorded values of 0) will be treated as <4.5 CFU/ml, i.e. non-detectable measurements will be treated as left-censored longitudinal observations in the analysis as we have done previously ^8^.

Based on this model, EFA will be compared between the two treatment arms in all patients (intention to treat), in the per-protocol population, and subgroups defined by HIV status (uninfected; infected) and baseline fungal burden (<5 log _10_ CFU/ml; ≥5 log _10_ CFU/ml).

## Analysis of secondary endpoints

### Survival until 10 weeks after randomization

Overall survival will be visualized using Kaplan-Meier curves and modeled using the Cox proportional hazards regression model with stratification by HIV status. In addition, survival will be modeled with a multivariable Cox regression model including the following covariates in addition to the treatment group: baseline log _10_-fungal load, Glasgow coma score less than 15 (yes or no), HIV infection status, and ARV treatment status at study entry (naïve or experienced).

### Disability at 10 weeks.

The disability score at week 10 follow-up is defined as the higher (worse) of “ the two simple questions” and the Rankin score assessed at that time point, and will be categorized as good outcome, intermediate disability, severe disability, or death (in case the patient died before 10 weeks) as previously described ^45^. The ordinal 10-week score (“good”>”intermediate”>”severe”>”death”) will be compared between the two arms with a proportional odds logistic regression model depending on the treatment arm and HIV infection status. The result will be summarized as a cumulative odds ratio with corresponding 95% confidence interval and p‐value. Patients lost to follow up will be analyzed according to their last recorded disability status. If the rate of patients lost to follow-up exceeds 10%, we will also perform an alternative analysis based on multiple imputation of missing values.

### Adverse events.

The frequency of serious and grade 3 & 4 adverse reactions as well as the frequency of specific adverse events will be summarized (both in terms of the total number of events as well as the number of patients with at least one event). The proportion of patients with at least one such event (overall and for each specific event separately) will be summarized and (informally) compared between the two treatment groups based on Fisher’s exact test.

### QT prolongation

The QTc will be classified as “normal” (<450ms for males, <460ms for females), mildly prolonged (≥450ms for males or ≥460 for females but ≤500ms) and prolonged (>500ms). The frequency of patients with a maximum QTc in these categories will be summarized for the following time intervals: day 1 (baseline), days 2-3, 4-7, 8-14, and after day 14. Comparisons will be with a proportional odds logistic regression model depending on the treatment arm and HIV infection status.

The maximum absolute change from baseline of the QTc during follow-up will also be summarized; the comparison between treatment groups will be based on a linear regression model with treatment as the main covariate and adjustment for HIV status and the baseline QTc interval.

### Analysis of other secondary outcomes

The rate of IRIS and the rate of relapse (defined as antifungal treatment intensification or re-treatment) will be modeled with cause-specific proportional hazards models with treatment as the only covariate and stratification by HIV infection status.

The visual acuity at 10 weeks is recorded on a 6 point scale and will be summarized by treatment arm for each eye separately, and overall where “overall” is defined as the worst recorded acuity of either eye. The odds of having “normal acuity” (amongst all surviving patients with a visual assessment) will be informally compared between the treatment arms with a logistic regression model adjusted for HIV status.

The time to the first new neurological event or death until 10 weeks will be analyzed in the same way as overall survival. Longitudinal measurements of intracranial pressure during the first 2 weeks will be modeled using a mixed effect model as described for the primary outcome. CD4 cell count at baseline and 10 weeks will be summarized and the change compared between the two groups based on a linear regression model with treatment as the main covariate and adjustment for HIV status and baseline CD4 cell count.

## Analysis populations

The primary analysis population for all analysis is the full analysis population containing all randomized patients except for those mistakenly randomized without cryptococcal meningitis. Patients will be analyzed according to their randomized arm (intention-to-treat). In addition the primary endpoint will be analyzed on the per-protocol population which will exclude the following patients: major protocol violations and those receiving less than 1 week of administration of the randomized study drug for reasons other than death.

## Interim analysis and role of the Data and Safety Monitoring Board (DSMB)

An independent DSMB will oversee the trial. Cardiac and unexpected serious adverse events with treatment allocation blinded will be reported to the DSMB within 10 days of occurrence and followed-up until resolution. The DSMB will perform an unblinded safety analysis after the first 20 patients have completed the allocated 2-week induction treatment or died. Particular attention will be paid to cardiac adverse events. Stopping for harm of Tamoxifen will be considered if a safety issue emerges which is sufficiently large, in the judgement of the DSMB, to suggest that continued exposure of patients to Tamoxifen is unethical. Early stopping for efficacy of Tamoxifen is not foreseen as this is a pilot study. The DSMB will be able to mandate additional safety analyses at any timepoint they deem fit.

At the interim analyses, the DSMB will receive a report including summaries of mortality, serious adverse events, grade 3 & 4 adverse events, and estimates of early fungicidal activity during the first 14 days by treatment arm. The report will be prepared by the DSMB statistician and distributed to all DSMB members for review. Based on these data, the committee will make recommendations on the continuation, cessation or amendment of the study. The study statistician will aid in setting-up the code for generating the interim analysis summaries in a blinded fashion, i.e. without access to the randomization assignment. The randomization list will be sent to the DSMB statistician directly from the study central pharmacist.

As the dissemination of preliminary summary data could influence the further conduct of the trial and introduce bias, access to interim data and results will be confidential and strictly limited to the involved independent statistician and the monitoring board and results (except for the recommendation) will not be communicated to the outside and/or clinical investigators involved in the trial.

Further reviews will be at the discretion of the DSMB. All DSMB reports, replies or decisions will be sent to the responsible Research Ethical Committees.

## Ethical considerations

### Declaration of Helsinki and Good Clinical Practice

The Investigator will ensure that this study is conducted in compliance with the current revision of the Declaration of Helsinki (Seoul 2008) and the Medical Research Council Guidelines on Good Clinical Practice (1998).

### Ethical Review

The study has been approved by the Oxford Tropical Research Ethics Committee (Reference 25-16), The ethics Committees of the Hospital for Tropical Diseases, Ho Chi Minh City (Reference CS/ND/17/07) and Cho Ray Hospital, Ho Chi Minh City (Reference 179 – CN-HDDD), and the Vietnam Ministry of Health (reference 3110/QD-BYT).

### Informed consent

The study staff will discuss the study with all potential adult participants or, in the case of a participant who is unable to give informed consent independently, with a representative appropriate within local culture. Study staff will describe the purpose of the study, the study procedures, possible risks/benefits, the rights and responsibilities of participants, and alternatives to enrolment. The participant or representative will be invited to ask questions which will be answered by study staff, and they will be provided with appropriate numbers to contact if they have any questions subsequently. If the participant or representative agrees to participate, they will be asked to sign and date an informed consent form (Appendix 5, Extended data ^58^). A copy of the form will be given to them to keep. If required, the participant or representative will be given up to 24 hours to consider the study provided the participant remains eligible for the study.

Participants who were consented by a representative will be approached to consider consent independently if at any time during study participation s/he becomes able to consider consent independently. 

In addition to the procedures above, illiterate signatories will have the informed consent form read to them in the presence of a witness who will sign to confirm that the form was read accurately and that the participant or representative agrees to participation. All patient information sheets and Consent/Assent forms will be written in the local language and will use terms that are easily understandable. Clinical care will not be delayed in any case during consideration of consent.

### Risks

This study will use a drug that has been studied thoroughly and its toxicities are well described. Further details can be found in the Study Treatment section of this protocol. While QT prolongation is described for Tamoxifen, life threatening arrhythmias have not been. QT prolongation has also been described for fluconazole. Polymorphic ventricular tachycardia has been described in association with fluconazole use, but this is an extremely rare event. It is not clear if combining Tamoxifen with fluconazole will increase the risk of prolonged QT interval and TdP. Less than 20% of cases of TdP will progress to ventricular fibrillation. The possible risk of TdP must be considered in the context of the risk of death of approximately 50% for patients with cryptococcal meningitis. Patients will be closely monitored for all adverse events and treated as per standard of care. Additional volumes of blood and cerebral spinal fluid will be taken for research tests. These volumes have very little risk of affecting the participant’s health. Some phlebotomy may be performed more often than is required by clinical care. This procedure carries the small risk of bruising and infection.

Tamoxifen and fluconazole may be teratogenic and Tamoxifen is present in breast milk. Therefore pregnant and breast-feeding women are excluded from this trial, and will be counselled to use contraception until 3 months after the study end.

### Benefits

It is unknown if study participants who receive study treatment will benefit. The additional monitoring and follow-up of patients by dedicated study staff may be of benefit to patients treated in resource limited settings. For the duration of the study all treatment costs associated with cryptococcal meningitis will be met including antifungal therapy, lumbar puncture, and study investigations. The study will pay for anti-arrhythmic drugs if needed, ECGs, and review by a cardiologist if clinically indicated. Training in laboratory and clinical procedures, research methods and good clinical practice, will be given to all participating centres. Investigators will engage with the HIV/AIDS community in each setting to ensure that trial conduct is cohesive with local patient support services. 

### Alternatives to Study Participation

The alternative to participation in this study is routine care by the doctors in the hospital. Patients will be responsible for their own treatment costs as per local norms and hospital policy.

### Confidentiality

Participants will be assured that all information generated in this study will remain confidential. The trial staff will ensure that the participants’ anonymity is maintained. Participant’s names will be recorded at the time of enrolment to allow for their identification at follow-up visits. However identifiable information will be linked to stored data or samples only by a protected Master List. This list will not be shared outside the study staff. All documents will be stored securely and all reports or samples will be coded without identifying information. Direct access will be granted to authorized representatives from the host institution and the regulatory authorities, if applicable, to permit trial-related monitoring and inspections. 

### Withdrawal of participants from the study

Each participant has the right to withdraw from the study at any time. All patients who withdraw will be referred for treatment as per routine clinical care. The reason for withdrawal will be recorded in the CRF.

### Sample sharing and storage

Samples collected will be used for the purpose of this study as stated in the protocol and stored for future use in studies not yet conceived, which may include genetic studies. Consent will be obtained from subjects for genetic testing and for sample storage and/or shipment of specific samples to collaborating institutions for investigations that cannot be performed locally. Any proposed plans to use samples other than for those investigations detailed in this protocol will be submitted to the relevant ethics committees prior to any testing.

The participants will be identified only by a study specific participant number and/or code in any database. The name and any other identifying detail will NOT be included in any study data electronic file.

### Sponsorship and Insurance

The University of Oxford has appropriate insurance-related arrangements in place in respect of the University's role as research sponsor for this study (Contact: University of Oxford, Research Services, University Offices, Wellington Square, Oxford OX1 2JD, Tel +44 (0) 1865 282585)

### Trial Registration

The trial was registered at ClinicalTrials.gov ID: NCT03112031 on the 13 April 2017.

## Data

### Data collection and entry

Source documents will be generated during the study by the site study staff at participating institutions. Source documents include all original recordings of observations or notations of clinical activities, and all reports and records necessary for the evaluation and reconstruction of the clinical trial. Source documents include, but are not limited to, the subject’s medical records, research case record forms (paper or electronic), laboratory reports, ECG tracings, x-rays, radiologist’s reports, subject’s diaries and questionnaires, biopsy reports, ultrasound photographs, progress notes, pharmacy records, and any other similar reports or records of procedures performed during the subject’s participation in the study.

Access to applicable source documents is required for study purposes. The site investigators are responsible for maintaining any source documentation related to the study. Source documentation should support the data collected on the CRF when the CRF is not the original site of recording. Source documentation must be available for review or audit by the sponsor or designee and any applicable regulatory authorities.

Case Report Forms (CRFs) will be used as a data collection tool. The study team will transfer the information from the source documents onto the CRFs. CRFs may be used as source documents if they are the primary data collection tool for specified data as documented in written standard operating procedures. The site Investigators are responsible for maintaining accurate, complete and up-to-date records. These forms are to be completed on an ongoing basis during the course of the study by authorized individuals.

Corrections to paper CRFs must be initialed and dated by the person making the correction and must not obliterate the original entry. All CRFs should be reviewed by the designated study staff and signed as required with written or electronic signature, as appropriate.

Selected study members (study doctors or nurses) will be trained on how to enter all clinical data as source information from the CRFs and from laboratory source documents into an internet-based computerized data entry system called CliRes hosted by OUCRU Viet Nam. Data entry will occur simultaneously as data are being generated during the trial as soon as possible after the information is generated. Data may be manually entered or scanned and electronically uploaded dependent on available software. Source documents and electronic data will be verified according to the Trial Monitoring Plan.

Following study completion and the main analyses and publication of the study results, the study sub-datasets consisting of the patient data from particular recruiting sites will be available to the investigators from those sites.

### Record Retention

The trial will be conducted to Good Clinical Practice (GCP) in line with the UK medical Research Council guidelines. The investigator is responsible for retaining all essential documents as listed in the MRC guidelines on GCP. All essential documentation for all study subjects are to be maintained in original paper format by the investigators in a secure storage facility for a minimum of 3 years and as required by local regulations thereafter. All essential documentation will be converted from paper to electronic format (if required) and stored centrally for at least 10 years after the completion of the trial and as required by local regulations thereafter. All stored records are to be kept secure and confidential.

## Monitoring

The trial will be conducted in compliance with this protocol, Medical Research Council Guidelines of Good Clinical Practice and any applicable regulatory requirement(s). 

The study will be adequately monitored by the OUCRU or their designate. Monitors will visit the clinical research site to monitor all aspects of the study in accordance with the appropriate regulations and the approved protocol. The objectives of a monitoring visit will be: 1) to verify the existence of adequately signed informed consent documents for each enrolled subject; 2) to verify the prompt, complete and accurate recording of all monitored data points, and prompt reporting of all SAEs and unexpected SAEs; 3) to compare abstracted information with individual subjects’ records and source documents (subjects’ charts, case report forms, laboratory analyses and test results, physicians’ progress notes, nurses’ notes, and any other relevant original subject information); 4) to verify the supply and condition of the study drug and the accurate and secure assignment of randomization code; and 5) to ensure protection of study subjects, investigators’ compliance with the protocol, and completeness and accuracy of study records. The monitors also will inspect the clinical site regulatory files to ensure that regulatory requirements and applicable guidelines are being followed. During the monitoring visits, the investigator (and/or designee) and other study personnel will be available to discuss the study progress and monitoring visit.

## Dissemination of findings

Results of the study will be published in international peer reviewed journals, presented at national and international conference, and publicized on the OUCRU website and through OUCRU social media accounts. Any publication or presentation during the active phase of the study must have permission from the Investigators. The investigators will define the strategy for publication, resolve any problems of authorship and maintain the quality of publications. All publications will acknowledge the appropriate authors and funding sources according to normal academic practice. The investigators are the custodian of the data and specimens generated from this trial.

## Trial status

The trial started recruiting patients in November 2017 and finished enrollment in May 2018. Data analysis is on-going.

## Discussion

Current best treatment for cryptococcal meningitis, consisting of amphotericin combined with flucytosine for the initial two weeks of treatment, is life-saving but remains associated with unacceptably high death rates in the order of 30–40%. Moreover, flucytosine, while an old drug that is off –patent, remains poorly available and extremely expensive. Its high cost (10–15,000 USD per week in the USA) illustrates that narrow spectrum, *Cryptococcus*-specific drugs are never likely to be a solution for the vast majority of patients, because there are no economies of scale in either drug development or manufacture. For this reason, the repurposing of off-patent drugs that have frequent indications for common, unrelated conditions, holds the main hope of improving outcomes from this devastating disease. Tamoxifen fits this profile perfectly. This controlled trial will deliver initial efficacy and safety data to enable the field to determine whether tamoxifen should be trialed in a larger study powered to mortality. If successful, tamoxifen has the potential to transform care of cryptococcal disease since it is cheap, has stable costs driven by a common alternate diagnosis (breast cancer), and is widely available. 

## Data availability

### Underlying data

All data underlying the results are available as part of the article and no additional source data are required

### Extended data

Additional information regarding the protocol is available as Extended data from the Oxford University Research Archive (ORA).

The document contain the following –

Appendix 1 - Management of raised intracranial pressure

Appendix 2 - Amphotericin administration and complications

Appendix 3 - Presumptive and definitive criteria for AIDS defining events

Appendix 4 - Drugs associated with prolonged QTc and TdP which must not be coadministered with Tamoxifen during the first 2 weeks of this study.

Appendix 5 - Patient Information sheet and Informed Consent Form

ORA: Extended data. Extended data - Appendices - A randomised controlled trial of tamoxifen for cryptococcal meningitis. https://doi.org/10.5287/bodleian:NG5DwvJDN ^58^

Licence: CC0 (CC Zero) attribution
