# Supplementary material for: A randomized open label trial of tamoxifen combined with amphotericin B and fluconazole for cryptococcal meningitis
Source: Wellcome Open Res. 2019 Jan 22;4:8. [Version 1] doi: 10.12688/wellcomeopenres.15010.1 (PMC6381443; doi:10.12688/wellcomeopenres.15010.1)
Supplement: Peer review history

# Peer review: Reviewer response for version 1

**DOI:** 10.21956/wellcomeopenres.16375.r34627

**Published:** 2019-03-11

**Authors:** Peter R. Williamson

**Affiliations:** 1 Laboratory of Clinical Immunology and Microbiology (LCIM), National Institute of Allergy and Infectious Diseases (NIAID), National Institutes of Health (NIH), Baltimore, MD, USA

Thoughts:

 This protocol describes a phase 2, open label randomized trial to evaluate the efficacy of the combination of tamoxifen, amphotericin and fluconazole compared to amphotericin and fluconazole alone. The reasons to use tamoxifen as an adjunct are valid given its synergistic activity with amphotercin and fungicidal effects. Tamoxifen has also proven to be synergistic with fluconazole *in vivo*, but mortality was not assessed in the mouse models used in that study. 

 However, the primary endpoint chosen is the EFA, which is a controversial surrogate marker for all cause mortality in patients with cryptococcal meningitis. While there are no studies that have shown a worsened outcome with improved EFA, there are studies that haven’t shown an improved survival at 10 weeks despite showing improved EFA ^1^. This should be taken into consideration when further phases of this study are designed and all cause mortality should probably be the primary endpoint.

 Under the Background (paragraph 1 line 2), the annual death rate is mentioned as 625,000/year based on a study published in 2008. In a more recent study published in the Lancet in 2017, the annual death rate is now recorded as 181,000/year ^2^.

 Some relevant questions about the protocol:

- Has the potential risk for Qt prolongation been included and well explained in the consent?
- Is there a sense of how much tamoxifen in the serum is protein bound and how much is available to be utilized by tissues?

I have read this submission. I believe that I have an appropriate level of expertise to confirm that it is of an acceptable scientific standard, however I have significant reservations, as outlined above.

## References

[1] MolloySKanyamaCHeydermanRLoyseAKouanfackCChandaDMfinangaSTemfackELakhiSLesikariSChanAStoneNKalataNKarunaharanNGaskellKPeirseMEllisJChawingaCLontsiSNdongJBrightPLupiyaDChenTBradleyJAdamsJvan der HorstCvan OosterhoutJSiniVMapoureYMwabaPBicanicTLallooDWangDHosseinipourMLortholaryOJaffarSHarrisonT: Antifungal Combinations for Treatment of Cryptococcal Meningitis in Africa. *New England Journal of Medicine*.2018;378(11) : 10.1056/NEJMoa1710922 1004-1017 10.1056/NEJMoa1710922 29539274

[2] RajasinghamRSmithRParkBJarvisJGovenderNChillerTDenningDLoyseABoulwareD: Global burden of disease of HIV-associated cryptococcal meningitis: an updated analysis. *The Lancet Infectious Diseases*.2017;17(8) : 10.1016/S1473-3099(17)30243-8 873-881 10.1016/S1473-3099(17)30243-8 PMC581815628483415

# Peer review: Reviewer response for version 1

**DOI:** 10.21956/wellcomeopenres.16375.r34634

**Published:** 2019-02-19

**Authors:** William G Powderly

**Affiliations:** 1 Division of Infectious Diseases, Department of Medicine, Washington University School of Medicine, St. Louis, MO, USA

This is a straightforward and very well written protocol that examines the potential benefit of adding tamoxifen to amphotericin B and fluconazole for cryptococcal meningitis, using as an end-point the early fungicidal activity of the combination therapy. The primary outcome has been well accepted as an early indicator of clinical efficacy and has correlated with clinical outcomes in other trials,

 My only relevant issue is why the combination of amphotericin B plus fluycytosine was not chosen as a control. The authors do make the very relevant point that given the widespread unavailability of flucytosine there is a need to have effective alternative regimens. Nonetheless, it would be optimal if there was a control group of amphotericin plus flucytosine. This is particularity pertinent given recent attention to the lack of antifungals on the essential drugs lists ^1^. 

 One minor point. There is a definite unmet medical need for more effective, tolerated, and available therapies for CM. Reference 1 could be replaced with a more up-to-date estimate of the global burden - which is still a large number ^2^.

I have read this submission. I believe that I have an appropriate level of expertise to confirm that it is of an acceptable scientific standard.

## References

[1] LoyseABurryJCohnJFordNChillerTRibeiroIKoulla-ShiroSMghambaJRamadhaniANyirendaRAliyuSHWilsonDLeTOladeleRLesikariSMuzooraCKalataNTemfackEMapoureYSiniVChandaDShimwelaMLakhiSNgomaJGondwe-ChundaLPerfectCShroufiAAndrieux-MeyerIChanASchutzCHosseinipourMVan der HorstCKlausnerJDBoulwareDRHeydermanRLallooDDayJJarvisJNRodriguesMJaffarSDenningDMigoneCDohertyMLortholaryODromerFStackMMolloySFBicanicTvan OosterhoutJMwabaPKanyamaCKouanfackCMfinangaSGovenderNHarrisonTS: Leave no one behind: response to new evidence and guidelines for the management of cryptococcal meningitis in low-income and middle-income countries. *Lancet Infect Dis*.2018; 10.1016/S1473-3099(18)30493-6 10.1016/S1473-3099(18)30493-6 30344084

[2] RajasinghamRSmithRMParkBJJarvisJNGovenderNPChillerTMDenningDWLoyseABoulwareDR: Global burden of disease of HIV-associated cryptococcal meningitis: an updated analysis. *Lancet Infect Dis*.17(8) : 10.1016/S1473-3099(17)30243-8 873-881 10.1016/S1473-3099(17)30243-8 28483415PMC5818156

# Peer review: Reviewer response for version 1

**DOI:** 10.21956/wellcomeopenres.16375.r34633

**Published:** 2019-02-05

**Authors:** Joshua Rhein

**Affiliations:** 1 Infectious Diseases Institute of Makerere University, Kampala, Uganda; 2 University of Minnesota, Minneapolis, MN, USA

Ngan and colleagues have presented a well-written and detailed protocol of a phase II, open-label, randomized controlled trial designed to evaluate initial efficacy and safety data for adjunctive tamoxifen added to standard antifungal therapy for cryptococcal meningitis. As the authors point out, the most effective treatment combination with amphotericin and flucytosine remains unavailable in much of the world where the burden of cryptococcal meningitis is the greatest. As such, the identification of a novel drug effective against *Cryptococcus* would represent an important advance. Given the inherent challenges involved in *de novo* antifungal development, drug repurposing is an attractive approach for new antifungal discovery.

 This experienced research group should be commended for advancing tamoxifen, a widely accessible SERM used in breast cancer, into clinical trial status for cryptococcal meningitis. In pre-clinical studies, tamoxifen has demonstrated fungicidal activity against *Cryptococcus* when combined with fluconazole and appears synergistic with amphotericin. In this protocol, the authors outline a well-designed randomized study in Vietnam comparing standard induction therapy (amphotericin + fluconazole) to standard induction therapy with the addition of tamoxifen 300mg daily.  The primary outcome is EFA. Secondary outcomes include 10-week survival and disability, grade ≥3 AEs including QT prolongation and visual deficits, rates of paradoxical IRIS and relapse, changes in intracranial pressure and CD4 count, and concentrations of tamoxifen and fluconazole in blood and CSF. The goal of the study is to inform the decision to proceed to a phase III trial powered for mortality, which is planned if adjunctive tamoxifen is found to improve CSF fungal clearance.

 I have no specific amendments suggested to this publication, but would offer the following comments as the authors move forward through the study process:

 Background and Rationale: Well-reasoned with appropriate background provided, including rationale of tamoxifen as antifungal, existing pre-clinical data, pharmacology and adverse effects.  My only comment would be regarding headings (might be more of a publication error).  Adverse events should be bolded, while subsequent subsections should not.

 Regarding EFA as a primary endpoint: have the authors considered the possibility of an “efficacy ceiling” for EFA that masks any potential activity of tamoxifen when it comes fungal clearance? For example, fluconazole is effective for cryptococcosis outside the induction period (consolidation, maintenance, asymptomatic CrAg antigenemia), though no mortality benefit has ever been convincingly demonstrated for fluconazole as part of combination induction therapy. In other words, demonstrating efficacy of tamoxifen in this study might be difficult using EFA as primary endpoint given presence of potent regimen containing amphotericin. If no improvement in EFA is observed during this trial, would the idea of tamoxifen as treatment for *Cryptococcus *be abandoned? Perhaps there would still be room for evaluating outcomes outside of the induction period?

 Statistics: again on the topic of EFA…  I would agree with the authors decision to determine EFA based on a linear mixed-effects model including all LP timepoint collected, as this would provide the most accurate and sensitive EFA for purposes of comparison between the two study groups. However, have the authors considered including other methods of calculating EFA as well?  It may be helpful in the final analysis to also include general linear regression models and to include models that limit the LP timepoints used in calculations to day 1, 7, and 14. This would be useful in comparing EFA across other studies, and bring attention to the fact that standardization of EFA across cryptococcal meningitis studies needs to be prioritized.

 Trial status: I am excited to see the results of this study!  Just curious why the authors submitted the protocol for publication only after enrollment into the study has concluded?

I have read this submission. I believe that I have an appropriate level of expertise to confirm that it is of an acceptable scientific standard.

# Response

**Published:** 2019-02-06

**Authors:** Jeremy Day

**Affiliations:** Hospital for Tropical Diseases, Oxford University Clinical Research Unit, Vietnam

Many thanks for the thoughtful comments. Were agree, particularly within the light of the recently published ACTA trial, that it is possible that a drug may have mortality benefits that are not necessarily detectable through measurement of EFA. However, in general, increased rates of EFA are strongly associated at the population level with improvements in survival. Particularly, the combination of amphotericin with flucytosine, which appears synergistic *in vitro* for the majority (~80%) of clinical isolates (as we found for tamoxifen and amphotericin), delivers the steepest declines in CSF fungal load, so EFA seemed an appropriate endpoint for this combination at this juncture.  This this will be the first use of tamoxifen within humans with cryptococcal disease - we felt it imperative to generate these efficacy and safety data prior to any trial powered to mortality. However, all patients will be followed until 10 weeks after randomisation, although we will lack power to demonstrate and effect with this sample size.

Publication of the protocol after the commencement of study enrolment represents unfortunate inefficiency on the part of the PI. The protocol was or course peer reviewed prior to ethical submissions and funding.
